# The Prognostic Roles of Systemic Inflammatory Markers Before Abiraterone or Enzalutamide Therapy in Metastatic Castration-Resistant Prostate Cancer

**DOI:** 10.3390/jcm14186536

**Published:** 2025-09-17

**Authors:** Harun Muğlu, Erdem Sünger, Lamia Şeker Can, Jamshid Hamdard, Özgür Açıkgöz, Özcan Yıldız, Ömer Fatih Ölmez, Mesut Şeker, Ahmet Bilici

**Affiliations:** 1 Department of Medical Oncology, Faculty of Medicine, Istanbul Medipol University, 34815 Istanbul, Türkiye; erdem.sunger@medipol.edu.tr (E.S.); jamshid.hamdard@medipol.com.tr (J.H.); ozgur.acikgoz@medipol.com.tr (Ö.A.); ozcan.yildiz@medipol.com.tr (Ö.Y.); olmezof@gmail.com (Ö.F.Ö.); abilici@medipol.edu.tr (A.B.); 2 Department of Medical Oncology, Faculty of Medicine, Istanbul Bezmialem Vakif University, 34093 Istanbul, Türkiye; lamia.can@bezmialem.edu.tr (L.Ş.C.); mseker@bezmialem.edu.tr (M.Ş.)

**Keywords:** prostate cancer, abiraterone, enzalutamide, NLR, PLR, inflammation, survival, prognostic factor

## Abstract

**Objectives:** The objective of this study was to investigate the prognostic value of systemic inflammatory markers (SIMs)—namely, the neutrophil-to-lymphocyte ratio (NLR) and platelet-to-lymphocyte ratio (PLR)—on survival outcomes and treatment responses in patients with metastatic castration-resistant prostate cancer (mCRPC) receiving abiraterone (ABI) or enzalutamide (ENZA) therapy. **Methods:** In this two-center retrospective observational study, researchers analyzed clinical data from 106 patients diagnosed with mCRPC. The cut-offs for NLR and PLR were determined to be 2.83 and 156, respectively, and their effects on progression-free survival (PFS) and overall survival (OS) were evaluated using Kaplan–Meier and Cox regression analyses. Changes in SIMs before and after ABI/ENZA treatment were assessed using the Wilcoxon signed-rank test. **Results:** Lower NLR (≤2.83) and PLR (≤156) were significantly associated with longer PFS and OS; however, in multivariate analysis, only high PLR emerged as an independent adverse prognostic factor for OS (HR: 2.01; *p* = 0.026). Meanwhile, treatment response was an independent predictor of PFS, and no significant changes were observed in the mean platelet volume (MPV), platelet distribution width (PDW), or platelet–large cell ratio (P-LCR) after treatment. **Conclusions:** SIMs, such as NLR and especially PLR, may serve as practical and accessible tools for predicting survival in mCRPC patients; however, further prospective studies are warranted.

## 1. Introduction

Prostate cancer is the second most common malignancy among men and represents a major cause of cancer-related mortality worldwide [1]. The clinical course of the disease is highly heterogeneous [2]; while localized stages can be effectively managed with surgery or radiotherapy, advanced cases often require systemic therapy [3]. Due to the central role of androgens in the development and progression of prostate cancer, androgen deprivation therapy (ADT) is considered the standard first-line treatment [4]. ADT is typically achieved through pharmacological castration, using luteinizing hormone-releasing hormone (LHRH) agonists or antagonists, or through surgical orchiectomy, and it is initially effective in the majority of patients; however, over time, many cases develop resistance to androgen deprivation and progress to the so-called “castration-resistant” phase [5].

Metastatic castration-resistant prostate cancer (mCRPC) is defined as prostate cancer that progresses biochemically, radiologically, or clinically despite ADT, with evidence of distant metastasis; this stage represents the most aggressive form of the disease and is associated with significantly worse prognosis [6,7]. Continued tumor progression despite serum testosterone levels below 50 ng/dL suggests that tumor cells can activate androgen receptor (AR) signaling pathways via alternative mechanisms [8]. Systemic treatment options in this phase include chemotherapy (e.g., docetaxel, cabazitaxel), radioisotope therapies (e.g., 177Lu-PSMA), immunotherapies, and targeted agents [9,10].

In the past decade, next-generation agents targeting the androgen receptor (AR) signaling pathways, particularly abiraterone acetate and enzalutamide, have transformed the treatment landscape of mCRPC by demonstrating significant survival benefits in both clinical trials and real-world settings [11,12]. Abiraterone (ABI), a CYP17A1 inhibitor that suppresses androgen production from adrenal and testicular sources, has shown efficacy both before and after chemotherapy (COU-AA-302 and COU-AA-301) when combined with prednisone [13,14]. Enzalutamide (ENZA), a potent AR antagonist, blocks multiple steps of AR signaling and improved survival in both pre- and post-chemotherapy settings (PREVAIL and AFFIRM) [15,16]. Both of these agents are now widely used, especially in patients unfit for chemotherapy.

Beyond clinical parameters, an increasing number of immunological, histopathological, and molecular biomarkers are being explored for prognostic and predictive purposes. Recent evidence has shown that PD-L1 expression correlates with Gleason Grade Group (GG), particularly in GG5 and in tumors with cribriform morphology, and is associated with shorter biochemical recurrence-free survival [17]. Similarly, a variety of histopathological markers, such as neuropilin-1, STAT3, IL-17, CD15/CD15s, and PSMA, have been linked to tumor progression, therapeutic resistance, and metastasis, underscoring their potential roles in risk stratification [18].

At the diagnostic level, biopsy-derived Gleason scores frequently underestimate the final grade obtained at radical prostatectomy, with upgrading observed in nearly one-fifth of cases and linked to adverse outcomes, such as biochemical recurrence [19]; these findings highlight the limitations of sampling and the potential value of integrating novel prognostic markers.

Advanced imaging has also improved disease characterization. PSMA PET/CT has shown high accuracy (over 90%) in detecting nodal metastases, with excellent performance in intermediate-risk patients (GG2), but reduced sensitivity for high-risk disease (GG5, ductal variants). Importantly, a negative PSMA PET/CT does not exclude the need for extended pelvic lymph node dissection in such cases [20].

At the molecular level, alterations including PTEN loss, ERG rearrangements, AR splice variants (AR-V7), and DNA damage repair defects (e.g., BRCA1/2, ATM mutations) are increasingly recognized as prognostic and therapeutic biomarkers; these abnormalities not only portend aggressive clinical behavior, but also guide treatment selection, with predictive implications for PARP inhibitors, platinum chemotherapy, and immune checkpoint inhibitors [21].

In recent years, growing awareness of the role of systemic inflammation in the tumor microenvironment has spurred interest in the prognostic value of systemic inflammatory markers (SIMs), such as the neutrophil-to-lymphocyte ratio (NLR) and platelet-to-lymphocyte ratio (PLR) [22,23]. While neutrophils may promote tumor progression, lymphocytes play a central role in antitumor immunity; thus, any imbalance in these ratios may reflect a disturbed immune–inflammatory response. Due to their affordability, accessibility, and reproducibility, these markers are emerging as practical prognostic tools in clinical practice [24].

Cancer has been widely recognized as a state of chronic inflammation, and excessive systemic inflammatory activation is considered tumor-promoting. Several studies have investigated “upper thresholds” for SIM values, showing that a markedly elevated NLR (>5) or PLR (>210) is associated with significantly worse survival outcomes in prostate cancer patients [25,26,27]. The above observations suggest that, while modest variations may reflect host–tumor balance, sustained high inflammatory ratios signal detrimental shifts toward tumor progression, angiogenesis, and immune evasion; this biological rationale underscores the importance of evaluating SIM cut-offs in prognostic models.

Mechanistically, neutrophils can facilitate prostate cancer progression by secreting pro-angiogenic and proteolytic factors (e.g., VEGF, MMP-9, elastase) and by enhancing immunosuppression through the expansion of myeloid-derived suppressor cells. Platelets protect circulating tumor cells from immune surveillance and contribute to metastatic spread via TGF-β and VEGF release. In contrast, lymphocytes—particularly cytotoxic CD8^+^ T cells—mediate antitumor immunity by inducing tumor cell apoptosis and orchestrating immune surveillance. Accordingly, elevated NLR and PLR reflect a pro-tumor inflammatory milieu and impaired host immunity, both of which have been consistently linked with inferior outcomes in prostate cancer [26,27,28].

In this context, we aimed to evaluate the prognostic impact of SIMs—particularly NLR and PLR—assessed before ABI/ENZA therapy in patients with mCRPC, with respect to prostate-specific antigen (PSA) response, clinical response to treatment, and survival outcomes. We seek to contribute to the literature via this study by supporting the integration of low-cost, easily applicable prognostic indicators into clinical decision-making.

## 2. Materials and Methods

### 2.1. Study Design and Centers

This retrospective, two-center observational study was conducted to evaluate the prognostic roles of pre-treatment SIMs on treatment responses and survival outcomes in patients with mCRPC treated with ABI/ENZA. The study was carried out at the Departments of Medical Oncology of Istanbul Bezmialem University and Istanbul Medipol University Department of Medical Oncology.

### 2.2. Patient Selection

Patients diagnosed with mCRPC and treated with ABI or ENZA between January 2018 and December 2024 at either center were retrospectively reviewed; inclusion criteria were as follows:

(1) Histologically confirmed prostate adenocarcinoma;
(2) Castration-resistant disease, defined by biochemical, radiological, or clinical progression despite castrate levels of testosterone (<50 ng/dL);
(3) Initiation of at least one cycle of ABI or ENZA;
(4) Availability of baseline clinical variables, PSA data, and pre-treatment complete blood count parameters.

Patients were excluded if they lacked laboratory data from the pre-treatment phase or initial follow-up period, had concurrent malignancies, or were lost to follow-up. Since we aimed to assess SIMs in this study, patients with conditions that might confound inflammatory markers, such as active infections, advanced heart failure (NYHA Class III or above), chronic kidney disease, or autoimmune/rheumatologic disorders, were also excluded.

### 2.3. Data Collection

Data were obtained from electronic medical record systems and patient files. Collected variables included demographics (age), disease characteristics (Gleason score, prior treatments such as ADT or docetaxel, and the number of systemic treatment lines prior to ABI/ENZA), ECOG performance status (PS), treatment response (clinical assessment and changes in PSA), dates of progression and death, and SIMs measured at baseline and during the first post-treatment evaluation. Other collected parameters included complete blood count-derived markers, including mean platelet volume (MPV), platelet distribution width (PDW), and platelet–large cell ratio (P-LCR); SIMs, namely, NLR and PLR, were calculated from respective cell counts [29].

### 2.4. Laboratory and Radiological Assessments, Variable Definitions, and ROC Analysis

Baseline blood test results obtained prior to the initiation of ABI/ENZA therapy were retrospectively retrieved from electronic medical records, and only values documented in the absence of clinically evident infection were included. Complete blood counts, including neutrophil, lymphocyte, and platelet parameters, had been measured at the time of routine clinical care using an automated hematology analyzer (Sysmex XN-1000, Sysmex Corporation, Kobe, Japan) under standardized internal and external quality control procedures. Neutrophil-to-lymphocyte ratio (NLR) and platelet-to-lymphocyte ratio (PLR) were calculated from absolute neutrophil, lymphocyte, and platelet counts, while additional platelet indices, such as mean platelet volume (MPV), platelet distribution width (PDW), and platelet–large cell ratio (P-LCR), were also recorded.

PSA levels were also retrieved retrospectively, and PSA response was defined, according to Prostate Cancer Working Group 3 (PCWG3) criteria, as a ≥50% decline from baseline, confirmed with a subsequent measurement ≥3 weeks later, provided that no radiographic or clinical progression had occurred [30].

Radiological assessments (contrast-enhanced CT and bone scintigraphy, and PSMA PET/CT in selected cases) were likewise collected retrospectively. All scans had originally been interpreted in line with PCWG3 criteria as part of clinical practice [31]. For this study, reports were reviewed, and radiological progression was defined as the appearance of new lesions or enlargement of existing lesions; when ambiguity was present, consensus review was performed by two radiologists.

Receiver operating characteristic (ROC) curve analyses were conducted retrospectively to identify optimal cut-offs for NLR and PLR with respect to PFS. The Youden index was applied to define thresholds. The resulting performance metrics (AUC, sensitivity, specificity) are presented in the Results section [32]. The above methodological approach is consistent with prior retrospective evaluations of systemic inflammatory markers in prostate cancer.

### 2.5. Statistical Analysis

All statistical analyses were performed using IBM SPSS Statistics version 27.0. Continuous variables are presented as median (minimum–maximum) or mean ± standard deviation, and categorical variables as counts and percentages. The Wilcoxon signed-rank test was used for paired comparisons of pre- and post-treatment values. Progression-free survival (PFS) and overall survival (OS) probabilities were estimated using the Kaplan–Meier method, and differences between groups were compared using the log-rank test.

To identify independent prognostic factors for PFS and OS, variables (clinical covariates, NLR, PLR, ECOG PS, treatment response, etc.) were first evaluated in univariate analysis and then included in a Cox proportional hazards regression model. Results are reported as hazard ratios (HRs) with 95% confidence intervals (CIs). A two-sided *p*-value < 0.05 was considered statistically significant.

Cut-offs for NLR and PLR were determined via receiver operating characteristic (ROC) curve analysis using Youden’s index. The optimal thresholds identified in our cohort were 2.83 for NLR (AUC 0.62, sensitivity 59.8%, specificity 66.7%) and 156 for PLR (AUC 0.68, sensitivity 48.8%, specificity 85.7%); these values provided the best discrimination for PFS and were used for dichotomization in subsequent analyses.

Importantly, there are no universally accepted thresholds for NLR or PLR in mCRPC. Reported cut-offs vary widely in the literature, with NLR typically ranging between 2.14 and 5, while PLR ranges between 150 and 210. Such variability reflects differences in patient populations (e.g., chemotherapy-naïve vs. post-docetaxel, ECOG PS endpoints (OS, PFS, PSA-PFS), and methodological strategies (ROC/Youden, median split, or literature-based fixed cut-offs)) [24,33]. For example, researchers of the COU-AA-302 [34] trial applied an NLR threshold of 2.5, those of the PREVAIL [35] study analyzed NLR as a continuous covariate, and those of the CARD study [36] used the median NLR of 3.38 as a cut-off.

To ensure robustness, we also conducted sensitivity analyses using alternative cut-offs frequently reported in the literature (e.g., NLR 2.5–3.0; PLR 150–190); these analyses confirmed that the prognostic impacts of NLR and PLR remained consistent in both direction and magnitude. The ROC curves demonstrating the discriminatory performance of NLR and PLR are presented in the Results Section.

### 2.6. Ethical Approval

This study was conducted in accordance with the principles of the Declaration of Helsinki. Ethical approval was obtained from the Non-Interventional Clinical Research Ethics Committee of Istanbul Medipol University (Approval No: 1212; Date: 28 November 2024). The application was submitted as a multicenter protocol, explicitly covering both Medipol University Hospital and Bezmialem Vakif University Hospital, and the approval permitted the use of patient data from both institutions. Informed consent was obtained from all individual participants prior to inclusion. Patient identities were kept confidential, and data were anonymized prior to analysis.

## 3. Results

In total, 106 patients with mCRPC and a median age of 69 years were included; prior docetaxel exposure was reported in 63.2% of patients. In the castration-resistant setting, enzalutamide and abiraterone were the most common first-line therapies. Baseline characteristics are summarized in Table 1.

**Figure 1:**
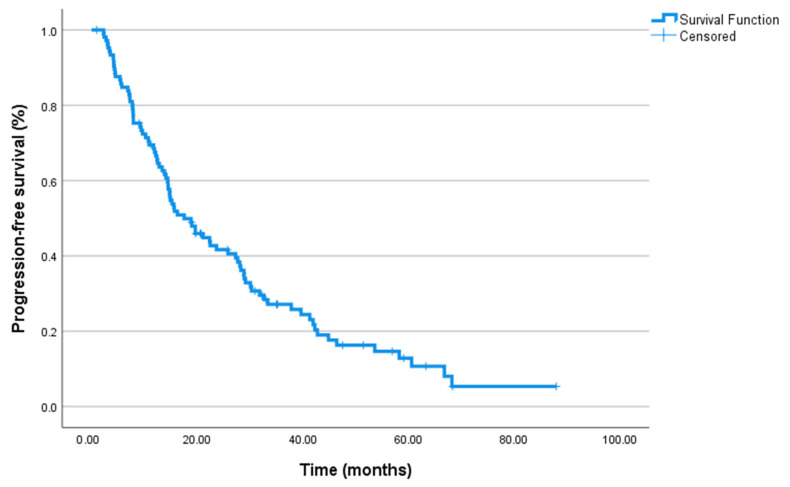
Kaplan–Meier curves for progression-free survival in the entire cohort (*n* = 106). Progression was defined according to PCWG3 criteria, based on PSA progression, radiographic progression on CT/bone scan (±PET/CT in selected cases), or clinical progression [30].

The median follow-up duration in the overall cohort was 28.3 months. During this period, PFS, as evaluated using Kaplan–Meier analysis, was calculated as a median of 17.5 months (95% CI: 11.99–23.07) for all patients. Similarly, the median OS was found to be 24.4 months (95% CI: 18.6–30.2). Kaplan–Meier survival curves for PFS are presented in Figure 1.

In subgroup analyses, treatment regimen (ADT alone vs. ADT plus docetaxel), prior docetaxel exposure, ECOG PS, and additional hematologic markers (MPV, PDW, and P-LCR) were not significantly associated with progression-free survival; these non-significant findings are summarized in Table 2.

**Figure 2:**
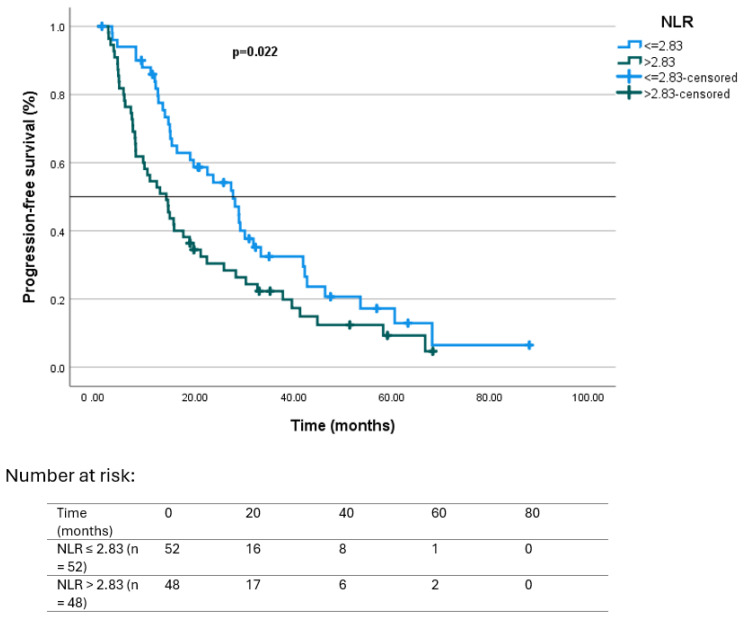
Kaplan–Meier survival curves according to baseline NLR category for PFS: NLR ≤ 2.83 (*n* = 51) vs. NLR > 2.83 (*n* = 55). Progression was defined according to PCWG3 criteria, based on PSA progression, radiographic progression on CT/bone scan (±PET/CT in selected cases), or clinical progression [30]. Hazard ratio (HR) for NLR >2.83 vs. ≤2.83 was 1.64 (95% CI 1.07–2.51, *p* = 0.022). Number at risk is shown below the *X*-axis.

**Figure 3:**
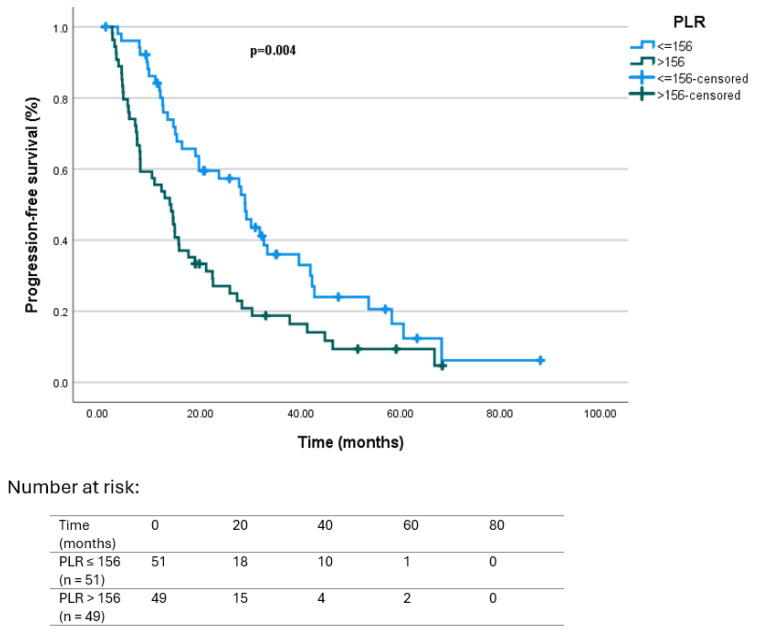
Kaplan–Meier survival curves according to baseline PLR category for PFS: PLR ≤ 156 (*n* = 52) vs. PLR > 156 (*n* = 54). Progression was defined according to PCWG3 criteria, based on PSA progression, radiographic progression on CT/bone scan (±PET/CT in selected cases), or clinical progression [30]. Hazard ratio (HR) for PLR > 156 vs. ≤156 was 1.89 (95% CI 1.21–2.95, *p* = 0.004). Number at risk is shown below the *X*-axis.

Regarding hematologic markers, patients with NLR ≤ 2.83 had a median PFS of 27.7 months (95% CI: 19.8–35.5), compared to 14.1 months (95% CI: 9.4–18.8) in those with NLR > 2.83 (*p* = 0.022). Similarly, median PFS was 28.9 months (95% CI: 25.9–31.9) in patients with PLR ≤ 156, and 13.8 months (95% CI: 9.3–18.3) in those with PLR > 156, with the difference reaching statistical significance (*p* = 0.022 and *p* = 0.004, respectively). Kaplan–Meier curves illustrating PFS according to baseline NLR and PLR categories are presented in Figure 2 and Figure 3, respectively.

In the overall cohort, 75 of 106 patients (70.8%) achieved a ≥50% decline in PSA levels (PSA50 response). When stratified by systemic inflammatory markers, the rate of PSA50 was significantly higher in patients with low baseline NLR (≤2.83) and PLR (≤156) compared with those above the thresholds. Patients with NLR ≤ 2.83 had a median PFS of 27.7 months versus 14.1 months for NLR > 2.83 (*p* = 0.022), while PLR ≤ 156 was associated with a median PFS of 28.9 months, compared with 13.8 months in patients with high PLR (*p* = 0.004). Similarly, median OS was significantly prolonged in patients with low NLR (37.1 vs. 18.8 months, *p* = 0.004) and low PLR (46.2 vs. 16.7 months, *p* < 0.001).

When PSA response and systemic inflammatory markers were evaluated together, patients with both PSA50 and low SIM levels (NLR ≤ 2.83 and/or PLR ≤ 156) had the most favorable outcomes, with a median PFS of 33.8 months and OS not reached during follow-up. In contrast, patients without PSA50 and with elevated SIM values had markedly inferior survival (median PFS 7.8 months, OS 10.6 months). Notably, among patients achieving PSA50, those with high PLR still had significantly shorter OS compared with those with low PLR, indicating that PLR adds prognostic information beyond biochemical response. In the multivariate analysis for OS, high PLR remained an independent adverse prognostic factor (HR: 2.01; 95% CI: 1.09–3.72; *p* = 0.026), whereas NLR lost significance.

In the analysis based on treatment response, patients who responded to therapy had a median PFS of 28.2 months (95% CI: 24.7–31.8), compared to 7.8 months (95% CI: 6.9–8.7) in non-responders. The corresponding PFS durations were 33.8 and 10.6 months, respectively, a difference which was statistically significant (*p* < 0.001), indicating that response to therapy is a strong predictor of PFS.

Regarding PSA levels, the median pre-treatment PSA was 9.00 ng/mL (range: 0–66,495), which decreased to 0.84 ng/mL (range: 0–51,508) after treatment, a change that was found to be statistically significant (Z = −6.283, *p* < 0.001), with 85.7% of patients demonstrating a meaningful decline in PSA levels.

In subgroup analyses, overall survival did not differ significantly according to treatment regimen (ADT alone vs. ADT plus docetaxel), line of systemic therapy in which ABI/ENZA was used, prior docetaxel exposure, or ECOG performance status; these insignificant findings are summarized in Table 2.

In a sensitivity analysis, restricted to patients who received abiraterone or enzalutamide as first-line therapy for mCRPC (*n* = 83), the associations of NLR and PLR with survival outcomes remained consistent with those observed in the overall cohort. Patients with PLR above the optimal threshold had significantly shorter overall survival, while no statistically significant impact on progression-free survival was noted. The above findings suggest that the prognostic effect of PLR on OS was not driven by treatment sequencing heterogeneity, but rather reflects an independent biological effect.

In the survival analysis based on baseline NLR, patients with NLR ≤ 2.83 had a median OS of 37.1 months (95% CI: 18.7–55.5), whereas those with NLR > 2.83 had a median OS of 18.8 months (95% CI: 13.5–24.1), with a statistically significant difference (*p* = 0.004). Similarly, in the analysis based on PLR, the median OS was 46.2 months (95% CI: 33.5–58.9) in patients with PLR ≤ 156, compared to 16.7 months (95% CI: 10.9–22.5) in those with PLR > 156; this difference was also statistically significant (*p* < 0.001). The above results suggest that elevated PLR and NLR may be associated with poorer prognosis.

In the overall cohort, the median OS was 24.4 months (95% CI: 18.6–30.2). Kaplan–Meier OS curves for the whole population (Supplementary Figure S1), stratified by NLR (Supplementary Figure S2), and stratified by PLR (Supplementary Figure S3) are provided in the Supplementary Materials.

**Figure 4:**
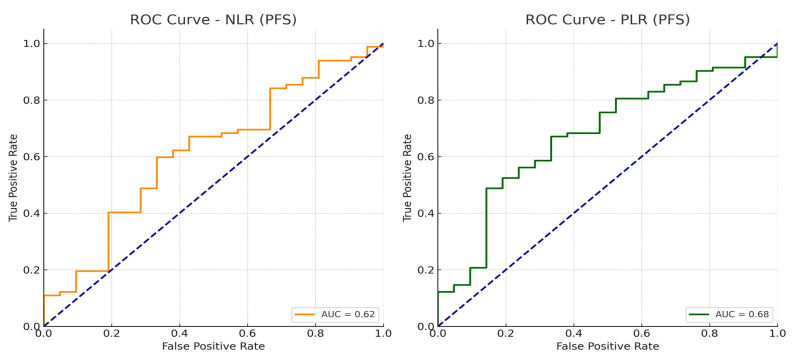
ROC curves for NLR and PLR predicting progression-free survival (PFS).

ROC curve analyses identified 2.83 for NLR and 156 for PLR as optimal cut-offs for predicting PFS (Figure 4).

In the multivariate Cox regression analysis for PFS, the model included PLR, NLR, ECOG PS, and treatment response. The overall model was statistically significant (χ^2^ = 49.609, df = 4, *p* < 0.001). Among the included variables, only treatment response was significantly associated with a lower risk of progression (hazard ratio (HR): 4.308, 95% CI: 2.634–7.046; *p* < 0.001). Although elevated PLR showed a trend toward increased risk of progression, it did not reach statistical significance (HR: 1.720, 95% CI: 0.967–3.061; *p* = 0.065). No significant associations were observed between PFS and NLR (*p* = 0.845) or ECOG PS (*p* = 0.475). The above findings suggest that, in multivariate analysis, treatment response was the only independent predictor of PFS.

In the multivariate analysis for OS, patients with PLR > 156 had significantly worse survival outcomes compared to those with PLR ≤ 156 (HR: 2.01, 95% CI: 1.09–3.72; *p* = 0.026). In contrast, in the NLR category, ECOG PS, and treatment response were not significantly associated with OS (*p* > 0.05). The above results indicate that PLR may serve as an independent prognostic factor for overall survival.

## 4. Discussion

In this study, we analyzed the prognostic impacts of SIMs—namely, NLR and PLR—on PFS and OS in patients with mCRPC, prior to treatment with ABI/ENZA. Our findings demonstrated that both NLR and PLR were significantly associated with survival outcomes, and, notably, elevated PLR was identified as an independent risk factor for shorter OS. Our results support the recently growing body of literature emphasizing the prognostic role of inflammation-based hematologic parameters. We selected data-driven cut-offs using ROC analysis and Youden’s index (NLR ≈ 2.63; PLR ≈ 175 for PFS discrimination). Notably, these thresholds fall within ranges commonly used in the literature (NLR around 3; PLR~130–190), which may facilitate external interpretability despite inter-study variability in assays and case-mix measurement. The fact that a PLR threshold optimized on PFS still predicted OS underscores the biological breadth of PLR beyond early disease kinetics, whereas NLR’s effect appears more sensitive to clinical context and competing covariates [37]. As shown in Figure 4, the ROC curves illustrate the discriminatory performance of these thresholds for PFS in our cohort, supporting their robustness and clinical applicability.

In our cohort, patients with lower pre-treatment NLR (<2.83) had significantly longer PFS, consistent with findings reported by Pisano et al., who showed that mCRPC patients with NLR > 3 experienced significantly shorter PFS and OS after ABI/ENZA therapy. Although NLR was significantly associated with PFS in univariate analysis in our study, this significance was lost in multivariate analysis, suggesting that the impact of NLR on clinical outcomes may depend on additional factors, such as PSA response or ECOG PS.

Importantly, when PSA response was evaluated together with systemic inflammatory markers, we observed that patients achieving both PSA50 and low SIM levels (NLR ≤ 2.83 and/or PLR ≤ 156) had the most favorable prognosis, whereas those without PSA50 and with high SIM levels had the worst outcomes. Notably, among PSA responders, patients with elevated PLR still showed significantly shorter OS compared with those with low PLR, indicating that PLR adds prognostic information beyond biochemical response; this observation aligns with prior meta-analyses demonstrating the adverse prognostic role of PLR in prostate cancer, but, to the best of our knowledge, no previous study has directly combined PSA response with SIMs to refine prognostic stratification [26,27,38]; therefore, our results suggest that integration of PSA and PLR may enhance risk assessment in daily practice.

With respect to NLR, several analyses have associated elevated baseline NLR with poor outcomes on AR-targeted agents, whereas cabazitaxel activity appears preserved, irrespective of NLR—an observation derived from the CARD study program and subsequent secondary analyses. Together with our findings (loss of independent effect for NLR on multivariable PFS/OS), this suggests that NLR may be more predictive of response to specific treatment classes, rather than purely prognostic across regimens, and supports the consideration of alternative strategies (e.g., taxanes) [39].

PLR, on the other hand, appeared to have a stronger prognostic significance in our analysis. Patients with PLR > 156 had significantly shorter OS (*p* = 0.006), and high PLR remained an independent prognostic factor in multivariate analysis (HR: 2.01; *p* = 0.026); this finding aligns with the meta-analysis by Guan et al., which included 11 studies and concluded that elevated PLR was associated with poor survival in mCRPC, with a particularly strong impact on OS [24].

Beyond statistical significance, the divergent behavior of PLR across endpoints is biologically plausible. Platelets facilitate tumor cell survival, immune evasion, and metastatic seeding through release of pro-tumorigenic mediators (e.g., TGF-β, VEGF) and physical cloaking of circulating tumor cells; such effects are more likely to influence long-term mortality than the early radiographic or PSA-based progression, as captured through PFS. Accordingly, multiple syntheses have reported a consistently stronger association of inflammatory ratios—particularly PLR—with overall survival than with short-term disease control, while also noting heterogeneity across studies and cut-off definitions. Our findings align with that pattern, with PLR independently prognostic for OS, but not for PFS, after multivariable adjustment [40].

Prior works in mCRPC treated with abiraterone or enzalutamide have variably emphasized NLR or PLR. For example, Pisano et al. observed that baseline NLR (≥3) was prognostic, whereas PLR was not considered in their cohort spanning pre- and post-docetaxel settings, highlighting between-study differences in patient mix and thresholds. In contrast, meta-analyses pooling multivariable results across disease stages or treatment lines have supported adverse prognostic associations for elevated PLR, especially for OS, albeit with substantial heterogeneity. Our data contribute to the literature by showing a robust, treatment line-independent association between PLR and OS in a contemporary ABI/ENZA-treated mCRPC population [37].

Similarly, Salciccia et al. highlighted the prognostic value of both NLR and PLR in a comprehensive meta-analysis that included both metastatic and non-metastatic prostate cancer patients; however, they emphasized that these markers had a more pronounced prognostic role in the metastatic subgroup [33]. Our study, which focused solely on mCRPC patients, further supports the clinical relevance of PLR within this population.

Interestingly, in our study, patients who responded to treatment had significantly longer PFS and OS. In multivariate analysis, treatment response emerged as the only independent predictor of PFS, highlighting the critical influence of early therapeutic response on survival and suggesting that biological response markers (e.g., inflammatory indices) may not be sufficient as standalone prognostic tools, an observation in line with subgroup findings from the CARD trial, which reported that high NLR was associated with poor response to AR inhibitors including ABI/ENZA, and that switching to cabazitaxel may be more advantageous in such patients [39].

Moreover, Guan et al. reported that PLR might be a more stable marker than NLR, which can be affected by infections, steroid use, and various immune conditions [24]; these biological differences may explain the stronger prognostic value of PLR observed in our study.

In another study by Fukuokaya et al., which examined PLR, along with other platelet parameters, high MPV was associated with poor prognosis in patients treated with ABI, but this association was not observed with ENZA [41], suggesting that inflammation-related markers may have treatment-specific prognostic implications. To address potential confounding due to treatment sequencing, we performed a sensitivity analysis restricted to patients receiving ABI/ENZA as first-line therapy for mCRPC. The direction and magnitude of associations were consistent with the overall cohort: higher PLR remained associated with worse OS, while no statistically significant impact on PFS emerged. The above results argue against treatment-line heterogeneity as the sole driver of the PLR–OS signal and support PLR as a line-agnostic prognostic marker in ABI/ENZA-treated mCRPC. Although we did not conduct separate analyses for ABI and ENZA in this study, due to limited sample size, PLR was found to hold prognostic value regardless of treatment agent.

In contrast, other platelet indices (MPV, PDW, P-LCR, MPV/PDW ratio) did not demonstrate significant prognostic associations or dynamic changes during treatment, in line with prior reports, which indicates that these inflammatory markers carry prognostic information primarily in the pre-treatment period and remain relatively stable throughout therapy; this may reflect that these indices are systemic manifestations of tumor-related inflammation and that hormonal therapies, such as ABI/ENZA, do not exert direct suppressive effects on these hematologic reflections. Similarly, Fukuokaya et al. found that platelet indices, including MPV and PDW, had prognostic significance at baseline, but did not change significantly during treatment with ABI/ENZA. Platelet indices may also exhibit treatment-specific prognostic behavior. Fukuokaya et al. reported that higher pre-treatment MPV portended poorer outcomes with abiraterone, but not with enzalutamide, implying mechanistic differences in how AR-directed agents intersect with platelet biology. Although our study was underpowered for drug-specific subgrouping, the sustained prognostic role of PLR across ABI/ENZA supports a platelet–inflammation axis of risk that is not confined to a single agent [41].

The above findings suggest that inflammation-based hematologic markers may be better interpreted as static prognostic indicators, rather than dynamic biomarkers. On the other hand, the lack of post-treatment changes might indicate that AR inhibitors have no direct anti-inflammatory effects in this patient population. Therefore, the prognostic and predictive roles of changes in inflammatory markers post-treatment remain controversial and require validation in larger, prospective studies.

Finally, a meta-analysis by Zhou et al. demonstrated that patients with high inflammatory markers had higher recurrence rates, even after radical treatments, suggesting that inflammation is a biologically relevant prognostic mechanism across all stages of disease [38].

This study is limited by its retrospective design, two-center setting, and modest sample size, which constrain adjustment for steroid exposure, intercurrent infections, antiplatelet use, and other blood count modifiers. Cut-off optimization was performed within the cohort and primarily on PFS; thus, external validation and time-dependent modeling are warranted. Given heterogeneous results across studies and endpoints, prospective, harmonized analyses incorporating composite inflammation scores (e.g., SII, PNI) and dynamic on-treatment changes may better delineate the relative prognostic versus predictive roles of NLR and PLR in mCRPC [42].

In our study, cut-offs of 2.83 for NLR and 156 for PLR were identified through ROC curve analysis using Youden’s index, providing the most accurate discrimination for progression-free survival in our cohort. The thresholds we used fall within the ranges most frequently reported in previous studies (NLR~2.14–5; PLR~150–210); however, a single universally accepted threshold does not exist, and considerable heterogeneity has been observed across studies, variability which can be attributed to differences in patient populations (e.g., chemotherapy-naïve vs. post-docetaxel, PSA burden, ECOG performance status), endpoints (OS, PFS, PSA-PFS), and statistical strategies (ROC-based, median-based, or fixed cut-offs derived from prior publications).

For instance, the COU-AA-302 trial [34] reported worse OS in patients with NLR > 2.5 treated with abiraterone, while the PREVAIL trial [35] demonstrated that NLR, modeled as a continuous covariate, was independently associated with OS. Similarly, in the CARD study [36], the median baseline NLR of 3.38 was used as a cut-off, and high NLR predicted inferior outcomes with abiraterone/enzalutamide, but not with cabazitaxel. Furthermore, meta-analyses have consistently highlighted the prognostic roles of NLR and PLR in prostate cancer, despite methodological heterogeneity across studies.

Taken together, the above findings suggest that, while different thresholds have been applied, the adverse prognostic implications of elevated NLR and PLR remain consistent. The ROC-derived cut-offs used in our study not only aligned with reported ranges, but also ensured optimal prognostic discrimination for our specific dataset, thereby reinforcing the robustness and clinical relevance of our results.

Additionally, our findings indicate that inflammation-based markers, which are low-cost, widely accessible, and easily integrated into clinical practice, have prognostic value in the mCRPC setting; however, prospective studies with larger sample sizes and more homogeneous patient populations are warranted before these markers can be routinely used to guide clinical decision-making.

## 5. Conclusions

In this study, the prognostic value of SIMs measured before ABI/ENZA treatment was evaluated in patients with mCRPC. The findings demonstrated that PLR was a significant predictor of OS. PLR appears to be a low-cost and clinically applicable prognostic marker that can be assessed prior to treatment. While NLR was significantly associated with PFS in univariate analysis, it did not retain independent prognostic significance in multivariate analysis.

In our study, patients who responded to treatment had a significantly longer PFS, and treatment response emerged as the strongest predictor of PFS in multivariate analysis, suggesting that achieving an early clinical response plays a central role in determining disease trajectory; on the other hand, no significant changes were observed in MPV, PDW, or P-LCR after ABI/ENZA treatment, indicating that these inflammation-based markers may serve as static prognostic tools, rather than dynamic indicators of treatment response.

Overall, the findings suggest that SIMs such as NLR and PLR, particularly when assessed at baseline, may have potential in risk stratification and personalized treatment planning. Despite the increasing use of molecular biomarkers, these parameters offer practical and economic advantages due to their simplicity and accessibility.

However, this study has certain limitations, including its retrospective design, relatively small sample size, and heterogeneous follow-up duration. Further prospective, multicenter studies with larger cohorts are needed to confirm the clinical applicability of our findings.

In conclusion, simple hematologic parameters, such as NLR and especially PLR, may provide meaningful prognostic information in patients with mCRPC and may contribute to the development of individualized treatment strategies in this population.

## Figures and Tables

**Table 1:** Baseline characteristics of the patients (*n* = 106).

Variable	Value
Age at diagnosis (years)	69 (40–90)
PSA at diagnosis (median, ng/mL)	82 (4–214,404)
PSA before ABI/ENZA (median, ng/mL)	2.84 (0.84–14.84)
Gleason score	
4 + 5	44 (41.5%)
4 + 4	22 (20.8%)
5 + 5	13 (12.3%)
4 + 3	10 (9.4%)
3 + 4	7 (6.6%)
Treatment in castration-sensitive phase	
ADT	56 (52.8%)
Docetaxel + ADT	50 (47.2%)
First-line treatment in CRPC phase	
Enzalutamide	51 (48.1%)
Abiraterone	32 (30.2%)
Docetaxel	18 (17.0%)
Cabazitaxel	3 (2.8%)
Lutetium	2 (1.9%)
Prior docetaxel before ABI/ENZA	
Yes	67 (63.2%)
No	39 (36.8%)
ECOG PS	
0	21 (19.8%)
1	82 (77.4%)
2	3 (2.8%)
Number of prior treatments before ABI/ENZA	
1 line	83 (78.3%)
2 lines	23 (21.7%)
Treatment response	
Yes	75 (70.8%)
No	31 (29.2%)
Clinical response	
Complete response	16 (15.1%)
Partial response	59 (55.7%)
Stable disease	13 (12.3%)
Progression	18 (17.0%)
PSA response	
Yes	75 (70.8%)
No	31 (29.2%)
NLR	
≤2.83	51 (48.1%)
>2.83	55 (51.9%)
PLR	
≤156	52 (49.1%)
>156	54 (50.9%)
MPV before ABI/ENZA (median, fL)	9.7 (9.2–10.4)
MPV after ABI/ENZA (median, fL)	9.7 (9.2–10.4)
PDW before ABI/ENZA (median, fL)	11.2 (10.5–12.1)
PDW after ABI/ENZA (median, fL)	11.3 (10.6–12.0)
P-LCR before ABI/ENZA, %	156.1 (IQR 120–195)
P-LCR after ABI/ENZA, %	156.1 (IQR 120–196)

Abbreviations: ABI: abiraterone, ADT: androgen deprivation therapy, ECOG PS: Eastern Cooperative Oncology Group Performance Status, ENZA: enzalutamide, MPV: mean platelet volume, NLR: neutrophil-to-lymphocyte ratio, PDW: platelet distribution width, PLR: platelet-to-lymphocyte ratio, P-LCR: platelet–large cell ratio, PSA: prostate-specific antigen. Notes: Clinical response was defined according to PCWG3 criteria [30]: complete or partial response, stable disease, or progression, based on PSA dynamics, radiological evaluation, and clinical findings. PSA response was defined as a ≥50% decline from baseline, confirmed with a subsequent measurement ≥3 weeks later. Thresholds for NLR (2.83) and PLR (156) were determined using ROC curve analysis with Youden’s index for PFS discrimination.

**Table 2:** Univariate analysis results for progression-free survival and overall survival.

Variable	Median PFS (Months) (95% CI)	*p*-Value (PFS)	Median OS (Months) (95% CI)	*p*-Value (OS)
Overall cohort	17.5 (11.99–23.07)	–	24.4 (18.6–30.2)	–
First-line treatment		0.618		0.884
ADT only	19.6 (12.6–26.7)		22.0 (17.3–26.7)	
Docetaxel + ADT	14.8 (9.0–20.6)		27.6 (17.8–37.4)	
Prior docetaxel use		0.896		0.744
Not administered	19.6 (7.4–31.8)		22.03 (16.93–27.14)	
Administered	15.6 (10.5–20.6)		24.97 (18.45–31.49)	
ECOG PS		0.963		0.498
ECOG PS 0	18.8 (1.3–36.4)		37.03 (13.44–60.62)	
ECOG PS 1	17.5 (11.6–23.5)		22.03 (17.11–26.96)	
ECOG PS 2	22.4 (9.7–35.2)		0.24 (0.00–48.57)	
NLR		0.022		0.004
NLR ≤ 2.83	27.67 (19.79–35.54)		37.1 (18.72–55.48)	
NLR > 2.83	14.07 (9.36–18.77)		18.83 (13.52–24.15)	
PLR		0.004		<0.001
PLR ≤ 156	28.87 (25.88–31.86)		46.2 (33.5–58.9)	
PLR > 156	13.80 (9.32–18.28)		16.7 (10.9–22.5)	
Treatment response status		<0.001		
Response	28.23 (24.67–31.80)		-	-
No response	7.83 (6.92–8.74)		-	-
PSA response status		<0.001		
Response	28.2 (24.7–31.8)		-	-
No response	7.8 (6.9–8.7)		-	-
Line of systemic therapy				0.637
ABI/ENZA as first line	-		25.7 (16.8–34.5)	
ABI/ENZA in second line or later	-		31.6(12.3–35.5)	
MPV ≤ 9.7 vs. >9.7 fL	17.0 (12.0–22.0) vs. 17.5 (11.5–23.5)	0.74	23.0 (17.0–29.0) vs. 24.0 (18.0–30.0)	0.62
PDW low vs. high	16.8 (11.2–22.4) vs. 18.0 (12.3–23.7)	0.84	22.5 (17.0–28.0) vs. 23.5 (18.5–29.0)	0.77
P-LCR low vs. high	16.9 (11.5–22.3) vs. 17.8 (12.7–23.9)	0.46	23.1 (17.5–28.7) vs. 24.2 (18.9–29.5)	0.55

Abbreviations: ABI: abiraterone, ADT: androgen deprivation therapy, ECOG PS: Eastern Cooperative Oncology Group Performance Status, ENZA: enzalutamide, CI: confidence interval, OS: overall survival, NLR: neutrophil-to-lymphocyte ratio, PLR: platelet-to-lymphocyte ratio, PSA: prostate-specific antigen, PFS: progression-free survival, MPV: mean platelet volume, PDW: platelet distribution width, P-LCR: platelet-large cell ratio.

## Data Availability

The data in this study are available from the corresponding author upon reasonable request.

## Supplementary Materials

The following supporting information can be downloaded at: https://www.mdpi.com/article/10.3390/jcm14186536/s1, Figure S1: Kaplan–Meier overall survival (OS) curve for the entire cohort (all patients); Figure S2: Kaplan–Meier OS curve stratified with NLR (≤2.83 vs. >2.83); Figure S3: Kaplan–Meier OS curve stratified with PLR (≤156 vs. >156).
