# Supplementary material for: The Prognostic Roles of Systemic Inflammatory Markers Before Abiraterone or Enzalutamide Therapy in Metastatic Castration-Resistant Prostate Cancer
Source: J Clin Med. 2025 Sep 17;14(18):6536. doi: 10.3390/jcm14186536 (PMC12470515; doi:10.3390/jcm14186536)
Supplement: Supplementary file 1 [file jcm-14-06536-s001.zip › jcm-3826810-supplementary.pdf]

Supplementary Materials

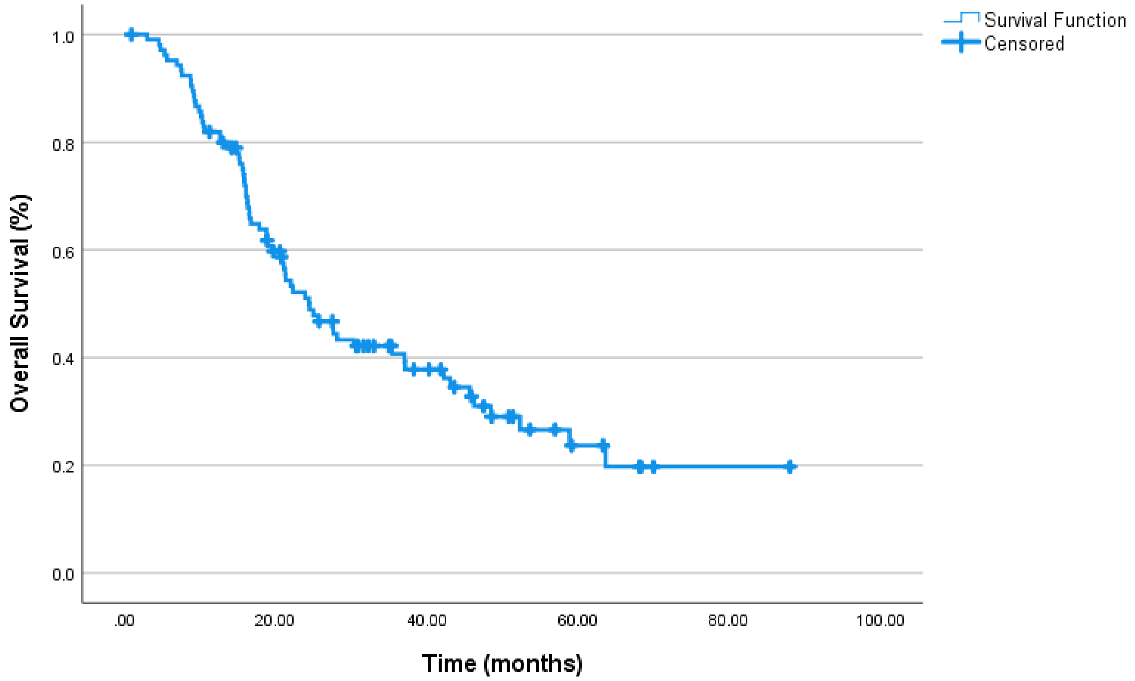

Supplementary Figure S1. Kaplan–Meier overall survival (OS) curve for the entire cohort (all patients).

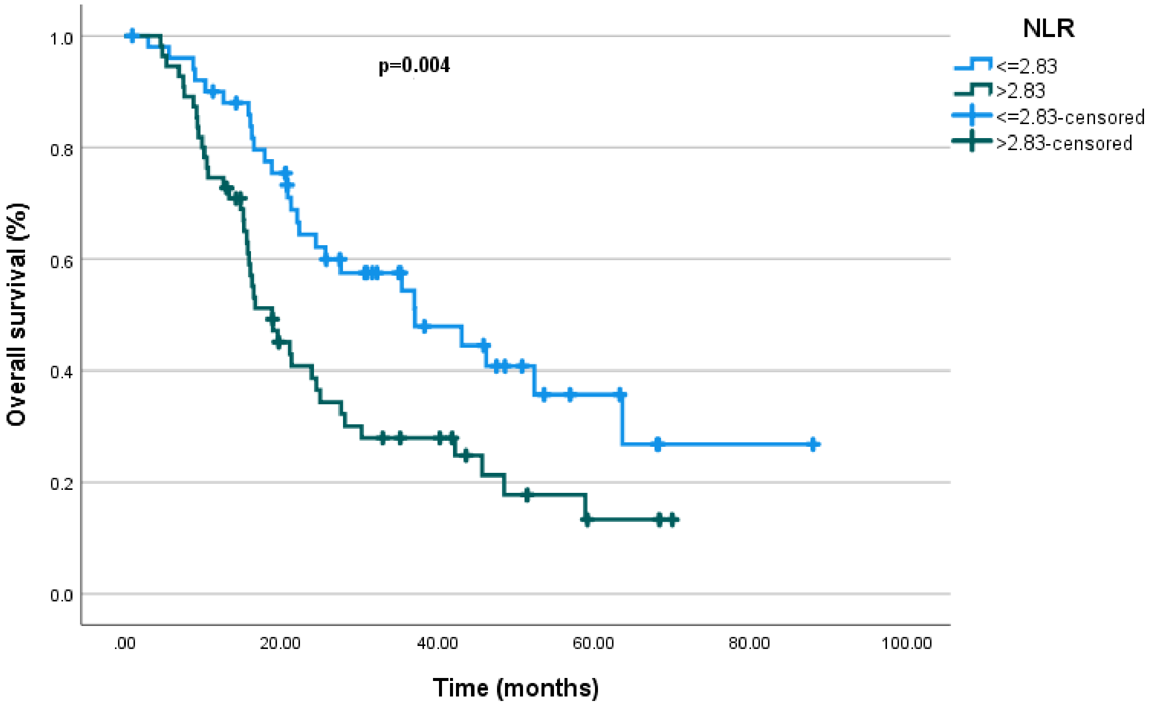

Supplementary Figure S2. Kaplan–Meier OS curve stratified with NLR (≤2.83 vs >2.83).

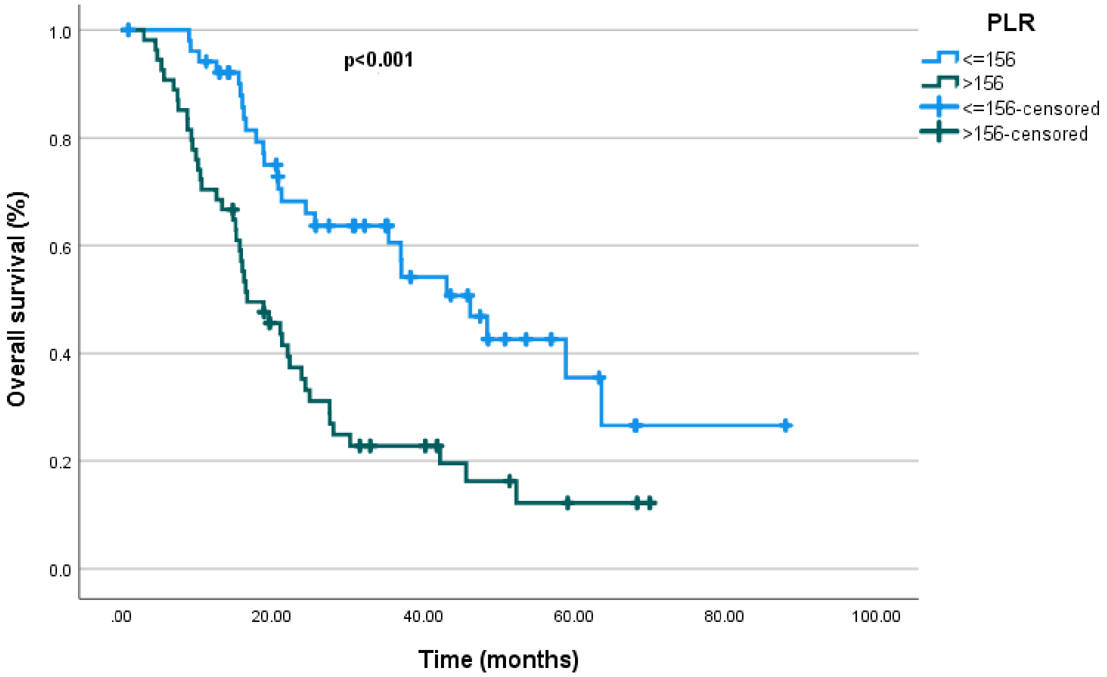

Supplementary Figure S3. Kaplan–Meier OS curve stratified with PLR (≤156 vs >156).
